# Treatment of Refractory Postdural Puncture Headache after Intrathecal Drug Delivery System Implantation with Epidural Blood Patch Procedures: A 20-Year Experience

**DOI:** 10.1155/2016/2134959

**Published:** 2016-08-11

**Authors:** Markus A. Bendel, Susan M. Moeschler, Wenchun Qu, Eugerie Hanley, Stephanie A. Neuman, Jason S. Eldrige, Bryan C. Hoelzer

**Affiliations:** ^1^Mayo Clinic, Rochester, MN 55905, USA; ^2^Ozark Orthopaedics, Fayetteville, AR 72703, USA; ^3^Gundersen-Lutheran Health System, La Crosse, WI 54601, USA

## Abstract

A recent publication reported the incidence of postdural puncture headache (PDPH) in conjunction with intrathecal drug delivery system (IDDS) implantation to be nearly 23 percent. Many patients responded to conservative measures but a percentage needed invasive treatment with an epidural blood patch (EBP). There is limited data to describe the technical details, success rates, and complications associated with EBP in this population. This study aims to provide a retrospective report of EBP for patients suffering from PDPH related to IDDS implantation. A chart review established a cohort of patients that required EBP in relation to a PDPH after IDDS implantation. This cohort was evaluated for demographic data as well as details of the EBP including technical procedural data, success rates, and complications. All patients received a trial of conservative therapy. Standard sterile technique and skin preparation were utilized with no infectious complications. The EBP was placed below the level of the IDDS catheter in 94% of procedures. Fluoroscopy was utilized in each case. The mean EBP volume was 18.6 cc and median time of EBP was day 7 after implant. There were no complications associated with EBP. EBP appears to be an effective intervention in this subset of PDPH patients.

## 1. Introduction

Intrathecal drug delivery system (IDDS) implantation can be an efficacious tool in managing patients with spasticity, chronic malignant or nonmalignant pain [1–9]. Many advances in our understanding of intrathecal medication administration and steady improvements in technology have helped to shape the best practices associated with device implantation and management [10]. However, this intervention is not without complication. Hematoma formation, neurologic injury, system failure, infection, catheter-associated granuloma formation, opioid induced hyperalgesia, continuous cerebrospinal fluid leak, seroma formation, and postdural puncture headache (PDPH) are known complications of IDDS placement [11–17].

A recently published retrospective review examining the incidence of PDPH in conjunction with IDDS implantation found symptoms in nearly 23 percent of patients [11]. The majority of these patients were managed conservatively with standard of care noninvasive measures including bedrest, intravenous hydration, caffeine supplementation, and analgesic medication administration [18, 19]. However, some patients continued to suffer severe symptoms of PDPH warranting an interventional management approach.

Epidural blood patch (EBP) is a well-described technique used to provide relief to patients suffering from resistant or severe PDPH. There is evidence to support its efficacy in relieving postdural puncture headache [20–23]. However, debate still remains regarding the necessity, effectiveness, timing, and exact technique of EBP [24, 25]. EBP carries a small risk of complications including unintended dural puncture, subarachnoid spread, hematoma formation, increased intracranial pressure, and neurologic injury [26–29]. However, the combination of the known risks of EBP and patients who have long-term intrathecal catheters in situ prompts new consideration. There are case reports describing the application of EBP or fibrin glue patch in patients with IDDS implants in the setting of continuous cerebrospinal fluid leak [30–33]. There is a scarcity of published material describing the outcomes, technical details, and complications associated with epidural blood patch in the PDPH population with IDDS in place. This provides a difficult landscape from which to ascertain the best practice in managing this small but important subset of patients with PDPH.

## 2. Objective

The objective of the current study was to provide a detailed review of management of refractory postdural puncture headache in patients with implanted IDDS who required epidural blood patch procedures over a 20-year time frame at a single academic tertiary care medical center (Mayo Clinic, Rochester, MN).

## 3. Methods

Following appropriate institutional review board (IRB) approval, we performed a retrospective cohort study based upon a 20-year chart review of clinical experience at a large academic medical center (Mayo Clinic, Rochester, MN). Patients who underwent placement of an IDDS during the period between June 1, 1989, and May 31, 2009, were included. The Mayo Clinic medical data retrieval service identified all patients who underwent implantation of an IDDS in this time frame with the use of ICD-9 codes. As described in the prior publication [11], a subset of patients who had symptoms consistent with PDPH was identified by examining all inpatient and outpatient clinical notes for a three-week period following IDDS implantation. This subset was examined for patients that proceeded to invasive intervention in the form of epidural blood patch. This formed the cohort.

The following data points were harvested from each medical record of the cohort: age at time of IDDS implantation, gender, indication for IDDS, date of IDDS implantation, methods of conservative PDPH management, IDDS catheter entry level, epidural blood patch level, epidural blood patch volume, interval between IDDS implantation and epidural blood patch, and preepidural blood patch skin preparation method. Improvement in headache symptoms after blood patch was recorded. Finally, each record was carefully screened for immediate and delayed post-EBP complications.

## 4. Results

The medical data retrieval service returned 319 surgical reports on 285 different patients over the specified time frame. As previously reported, chart review uncovered postdural puncture headache symptoms in 73 patients (22.9%) [11]. Of the patients with PDPH, 15 patients had symptoms refractory to conservative treatment and required 17 epidural blood patch procedures. The complete results are summarized in Table 1.

### 4.1. Conservative Treatments

All patients received a trial of at least one conservative measure. The conservative measures and their incidence of use in this cohort are as follows: bedrest (100%), intravenous hydration (67%), caffeine (67%), acetaminophen (47%), and antiemetic therapy (27%). See Table 2.

### 4.2. Infectious Precautions

All epidural blood patches were performed under standard sterile technique. Three cases did not have skin preparation explicitly documented (20%). All others were completed with chlorhexidine (60%), betadine (13%), or both (7%). One patient received prophylactic intravenous antibiotics due to proceduralist preference.

### 4.3. Image Guidance

All of the procedures were performed with fluoroscopic guidance to ensure proper needle positioning.

### 4.4. EBP Level

The blood patch was performed below the level of the IDDS catheter insertion in 94% of cases. See Table 3.

### 4.5. EBP Blood Volume

One procedure did not have volume administered explicitly recorded in the medical chart. The mean volume for the remaining procedures was 18.6 mL. The volume administered was determined by the development of patient reported paresthesias or a maximum volume of 20 mL.

### 4.6. EBP Timing

The initial blood patch was placed on median postimplantation day number 7. The range of initial blood patch timing ranged from 3 to 36 days after IDDS implantation. Two patients required repeat blood patch application—these occurred on days 15 and 28 after implant which were 3 and 8 days after the initial EBP, respectively. See Table 4.

### 4.7. PDPH Symptom Resolution

All patients reviewed achieved relief of the PDPH symptoms. First-time blood patch success rate was 10/15 (67%). Two patients required repeat blood patches that were both successful (13%). Three patients (20%) were found to have persistent cerebrospinal fluid leak that required reoperation with purse-string suture placement and/or surgical fibrin glue placement at the IDDS catheter entrance site.

### 4.8. Complications

We found no complications directly related to performing an epidural blood patch in this cohort.

## 5. Discussion

The goal of this study was to address the relative paucity of published technical data in placing an epidural blood patch in patients with refractory PDPH in the setting of a recently placed IDDS. As previously reported, the incidence of PDPH in this population is likely higher than initially believed [11]. Conservative measures are effective in relieving the underlying symptoms in the majority of cases. However, a portion of this population continues to have significant amount of PDPH symptomology that is deleterious to their quality of life. This series suggests that epidural blood patches can be performed in this population with a reasonable margin of safety and efficacy. We found no increased incidence of complications. There are, however, case reports in the literature of complications utilizing this technique in this population [34]. Given the minimal amount of data in the literature relating EBP, PDPH, and IDDS, it is difficult to estimate the true incidence of complications in this population compared with other PDPH and EBP populations.

There do not appear to be any technical nuances specific to performing an EBP in a patient with IDDS, other than avoiding direct trauma to the IDDS catheter in situ by utilizing fluoroscopy. This series would suggest that standard sterile technique with betadine or chlorhexidine skin preparation is satisfactory for infectious prophylaxis. Only one patient received intravenous prophylactic antibiotics and there were no infections observed in our dataset. It has been an effective practice to place the EBP from 1 to 4 interspaces below the site of the IDDS catheter. The volume administered varied according to development of patient reported paresthesias, but often the patient received the goal volume of twenty milliliters. The practice of performing an epidural blood patch has evolved to include higher volumes of administered blood due to data highlighting the importance of pressure paresthesia [28, 29]. Therefore, twenty milliliters may not have been an optimal volume in those patients who did not develop a pressure paresthesia. Despite this, a large majority of patients (80%) did report relief from one or two blood patches.

Epidural blood patching appears to be an effective intervention for patients who are afflicted with PDPH related to IDDS implantation. A majority of the patients obtained lasting relief after application of a single blood patch, while a small subset needed a second blood patch. This is consistent with success rates of EBP in other patient populations [22, 23]. Of the patients who did not respond initially, it appears reasonable to proceed with repeat EBP or epidural fibrin glue administration. On rare occasions, patients may require operative intervention with dural repair or revision. 

*Limitations*. This study has recognizable limitations. Although our dataset spans a time frame of 20 years, it includes only 15 patients in our cohort. It is difficult to extrapolate to an entire population based on this sample size. Secondly, a retrospective chart review study design is not sufficient to establish any cause-effect relationship, but it would be very difficult to construct a randomized, prospective trial to assess the effectiveness of this intervention. Lastly, this dataset includes a compilation of several proceduralists with varying preferences in their procedural technique. It is impossible to control for individual preference retrospectively and this makes the interpretation of the results more difficult.

## 6. Conclusions

In conclusion, this study has evaluated the largest known-to-date cohort of patients with PDPH after IDDS implant and provided a technical report on how these patients have been managed with EBP. EBP appears to be an effective intervention in this subset of PDPH patients and no complications were observed utilizing the techniques as outlined above. Repeat blood patches or epidural fibrin glue administration may be helpful for patients who do not respond to initial EBP.

## Figures and Tables

**Table 1:** Summary of results.

Patient	Age	Gender	Indication	Conservative treatments	Catheter level	EBP level	Image guidance?	Volume (mL)	POD#	Skin prep	Complications
1	54	M	Multiple sclerosis	1, 2, 3, 5	L1-2	L2-3	Yes	20	4	ChloraPrep	None
2	49	F	Ovarian cancer	1, 2, 3, 4, 5	L4-5	L5-S1	Yes	20	5	ChloraPrep	None
3	57	M	Multiple sclerosis	1	L2-3	L3-4	Yes	20	36	ChloraPrep	None
4	49	F	Spasticity; spinal cord injury	1, 2, 3, 4, 5, 6	L3-4	L4-5	Yes	12	13	ChloraPrep	None
5^*∗*^	54	M	Prostate cancer	1, 2, 3	T10-11	T10-11	Yes	15	16	Bacitracin	None
6	45	F	Multiple sclerosis	1	L2-3	L4-5	Yes	20	3	ChloraPrep	None
7^*∗∗*^	72	M	Lung cancer	1, 2	L1-2	L3-4 L5-S1	Yes	15 20	20 28	ChloraPrep	None
8	45	F	Breast cancer	1, 3, 5	L2-3	L4-5	Yes	20	8	ChloraPrep Betadine	None
9	43	M	Spasticity; spinal cord injury	1, 2, 3	L2-3	L4-5	Yes	20	7	ChloraPrep	None
10	49	M	Pancreatic cancer	1, 2, 3, 4	L3-4	L5-S1	Yes	20	3	ChloraPrep	None
11^*∗∗*^	30	M	Spasticity; spinal cord injury	1, 2, 3, 4, 5	L3-4	L4-5 L4-5	Yes	20 22	12 15	Betadine	None
12	34	F	Melanoma	1	L2-3	L3-4	Yes	18	14	Unknown	None
13	50	F	Pancreatic cancer	1, 2, 3, 5	L1-2	L5-S1	Yes	15	7	ChloraPrep	None
14	43	F	Multiple sclerosis	1, 2, 3, 5	L2-3	L3-4	Yes	20	6	Betadine	None
15	53	F	Multiple sclerosis	1	L3-4	L5-S1	Yes	Unknown	7	Unknown	None

Key: conservative treatments:

(1) Bedrest.

(2) IV fluids.

(3) Caffeine.

(4) Antiemetics.

(5) Acetaminophen.

(6) Opioids.

^*∗*^Patient found to have persistent CSF leak despite initial blood patching. Ultimately required reopening of the incision and placement of a purse-string stitch and/or fibrin glue along the catheter insertion site.

^*∗∗*^Transient relief after initial blood patch. Blood patch was repeated and symptoms resolved.

**Table 2:** Incidence of conservative treatments.

Number of conservative treatments employed	Incidence
1	27% (4/15)
2	7% (1/15)
3	20% (3/15)
4+	47% (7/15)

**Table 3:** Location of epidural blood patch.

Location of EBP relative to IDDS catheter	Incidence
At IDDS insertion site	6% (1/17)
1 level below	47% (8/17)
2 levels below	35% (6/17)
3 or more levels below	12% (2/17)

**Table 4:** Timing of epidural blood patch after IDDS implant.

Time of EBP after IDDS implant	Incidence
1–7 days	53% (8/15)
8–14 days	27% (4/15)
15+ days	20% (3/15)
